# The impact of mild, moderate, and severe visual field loss in glaucoma on patients’ quality of life measured via the Glaucoma Quality of Life-15 Questionnaire: A meta-analysis

**DOI:** 10.1097/MD.0000000000008019

**Published:** 2017-12-01

**Authors:** Yue Wang, Sameh Alnwisi, Min Ke

**Affiliations:** Ophthalmology of Zhongnan Hospital of Wuhan University, Wuhan, HuBei Province, China.

**Keywords:** glaucoma, Glaucoma Quality of Life-15 Questionnaire, quality of life, visual field

## Abstract

**Background:**

We performed a meta-analysis to determine the impact of mild, moderate, and severe visual field loss on quality of life (QoL) in patients with glaucoma.

**Methods:**

A comprehensive literature search was performed using the *PubMed*, *Excerpta Medica dateBASE* and *China National Knowledge Infrastructure* databases to identify relevant articles published before June 30, 2016. Patients’ QoL was measured using the Glaucoma Quality of Life-15 Questionnaire (GQL-15).

**Results:**

In total, 401 patients with glaucoma and 205 patients without glaucoma participated in 4 experiments. The GQL-15 summary scores are statistically different between patients with and without glaucoma (standard mean difference [SMD] = 0.94, 95% confidence interval [CI]: 0.73–1.16, *P* < .01). GQL-15 summary scores for patients with mild, moderate, and severe visual field loss all differed significantly from those of patients without glaucoma; the SMDs for their summary scores were as follows: mild: 1.24, 95% CI: 0.26 to 2.22, *P* = .01; moderate: 2.05, 95% CI: 0.91 to 3.19, *P* < .001; and severe: 2.57, 95% CI: 1.44 to 3.71, *P* < .001. Two factor scores for central and near vision (SMD = −0.35, 95% CI: −1.01 to 0.30, *P* = .29) and glare and dark adaptation (SMD = −0.36, 95% CI: −1.01 to 0.30, *P* = .28) did not differ significantly between patients with mild and moderate visual field loss. However, summary scores and 2 factor scores (peripheral vision and outdoor mobility) differed significantly between patients with mild and moderate glaucoma. In addition, summary scores and all 4 factor scores differed significantly between patients with mild and severe glaucoma. Moreover, summary scores and 3 factor scores (peripheral vision, glare and dark adaptation, and outdoor mobility) differed significantly between patients with moderate and severe glaucoma. However, scores for 1 factor (central and near vision) did not differ significantly between any of the patient groups (SDM = −0.53, 95% CI = −1.33 to 0.27, *P* = .19).

**Conclusions:**

Glaucoma patients with visual field loss showed significantly poorer QoL relative to that of patients without glaucoma. Patients’ QoL decreased as their glaucoma severity increased.

## 1 Introduction

Glaucoma is the second leading cause of blindness worldwide, and the number of people with bilateral blindness resulting from glaucoma is expected to exceed 11 million by 2020.^[1]^ Patients with glaucoma require life-long treatment, which is often altered according to disease development, and regular follow-up. Initial or revised glaucoma treatment seldom meets patients’ expectations. In addition, development of the disease could cause progressive reduction of visual acuity and loss of the visual field, increasing patients’ anxiety and fear of blindness. Moreover, visual disability and psychological pressure have been shown to exert a strong impact on quality of life (QoL) in patients with glaucoma.

Intraocular pressure (IOP), visual acuity, optic disc, and computerized perimetry have traditionally been used as clinical indicators in the assessment and management of glaucoma. However, clinicians’ awareness of the importance of QoL in patients with glaucoma has gradually increased; therefore, several studies have demonstrated a correlation between clinical indices for glaucoma (such as vision, the visual field, and visual function) and patients’ QoL.^[2–6]^ Of these clinical variables, visual acuity is the most important indicator of daily functioning.^[7]^ In addition, visual field has recently been strongly associated with QoL in glaucoma patients.^[8]^ Therefore, we screened for studies that used the Glaucoma Quality of Life-15 Questionnaire (GQL-15) to measure QoL, and performed a meta-analysis to compare the effects of mild, moderate, and severe visual field loss on QoL in patients with glaucoma.

## 2 Methods

### 2.1 Search strategy

Three electronic databases *PubMed*, *EMBASE*, and *CNKI* database were researched systematically for studies published. Searches were conducted using the key words “glaucoma,” “quality of life OR QoL,” and “Glaucoma Quality of life-15 OR GQL-15.” Three electronic databases, *PubMed*, the *Excerpta Medica database*, and the *China National Knowledge Infrastructure*, were searched systematically for studies published before June 30, 2016. Searches were performed using the following keywords: “glaucoma,” “quality of life OR QoL,” and “Glaucoma Quality of life-15 OR GQL-15.” *Google Scholar* was searched for additional information.

### 2.2 Inclusion and exclusion criteria

Studies that met the following criteria were included in the analysis: patients with glaucoma and a control group without glaucoma as participants; measurement of QoL in glaucoma patients using the GQL-15; classification of glaucoma patients into 3 groups according to disease stage, determined via examination of the visual field using perimetry; and original articles in English and Chinese. Reviews, longitudinal studies, study design protocols, studies that did not report mean scores with standard deviations (SDs) for summary scores and the 4 GQL-15 factors scores, and studies with participants in specific age groups (e.g., young or elderly glaucoma patients) were excluded from the analysis. In studies in which data were collected repeatedly from the same group of patients, only the most recent series were included, and data that could not be obtained from a final publication were obtained from previous reports.

### 2.3 Assessment of study quality

Study quality was assessed using the Newcastle–Ottawa Scale (NOS), which includes 3 categories (8 items) for case–control studies: selection (4 items), comparability (1 item), and exposure (3 items). Studies are awarded a maximum of 1 star for each numbered item in the selection and exposure categories and a maximum of 2 stars for each item in the comparability category. Studies with ≥5 stars were included in the meta-analysis. The NOS criteria for study quality were as follows—8 to 9 stars: high quality; 6 to 7 stars: medium quality; 5 stars: low quality.

### 2.4 Classification of glaucoma severity

Patients were classified into 3 groups according to the extent of their loss of the central visual field ^[9]^: “mild” (unilateral loss of less than half of the visual field), “moderate” (unilateral loss of more than half of the visual field or bilateral loss with less than half of the visual field lost in each eye), and “severe” (bilateral loss with more than half of the visual field lost in either eye).

### 2.5 GQL-15 questionnaire score

Patients’ QoL was measured using the GQL-15 questionnaire. The GQL-15 includes 15 items divided between 4 factors pertaining to visual disability: central and near vision, peripheral vision, dark adaptation and glare, and outdoor mobility. The GQL-15 items reflect each factor and are represented by a code between 0 and 5, as follows: 0, abstinence from activity for reasons unrelated to vision; 1, no difficulty; and 5, severe difficulty. The subscale score for each factor is calculated as the average of the sum of the item scores. Higher subscale scores indicate greater difficulty in performing vision-related activities and poorer QoL. The maximum summary score is 75, and higher scores indicate poorer QoL. In the present study, we used summary and factor scores as measures of QoL in glaucoma patients.

### 2.6 Data extraction

We collected the following data from all of the included studies: author, publication year, study design, number of participants, age (mean and SD or range), and outcome measures. We also extracted GQL-15 questionnaire summary scores and scores for the 4 factors (mean and SD) for all patients with glaucoma and patients with mild, moderate, and severe glaucoma. All analyses were based on previous published studies, thus no ethical approval and patient consent are required.

### 2.7 Statistical meta-analysis

Some of the trials did not report all of the outcomes of interest. Therefore, we performed separate meta-analyses for each comparison and outcome. The statistical analyses were performed using RevMan 5.3 software. Standardized mean differences (SMDs) were calculated for the GQL-15 scores, and 95% confidence intervals (CIs) were calculated for all outcomes. The significance level was set at *P* < .05. The statistical heterogeneity of the studies was assessed using a χ^2^ test with calculation of *I*^2^ (*I*^2^ < 50% and *P* > .1 might not be important, *I*^2^≥ 50% and *P* ≤ .1 were considered statistically significant heterogeneity). If there was heterogeneity between studies, the data were pooled and included in a random-effects model.^[10]^ Otherwise, a fixed-effect model was created.

## 3 Results

### 3.1 Literature search

**Figure 1:**
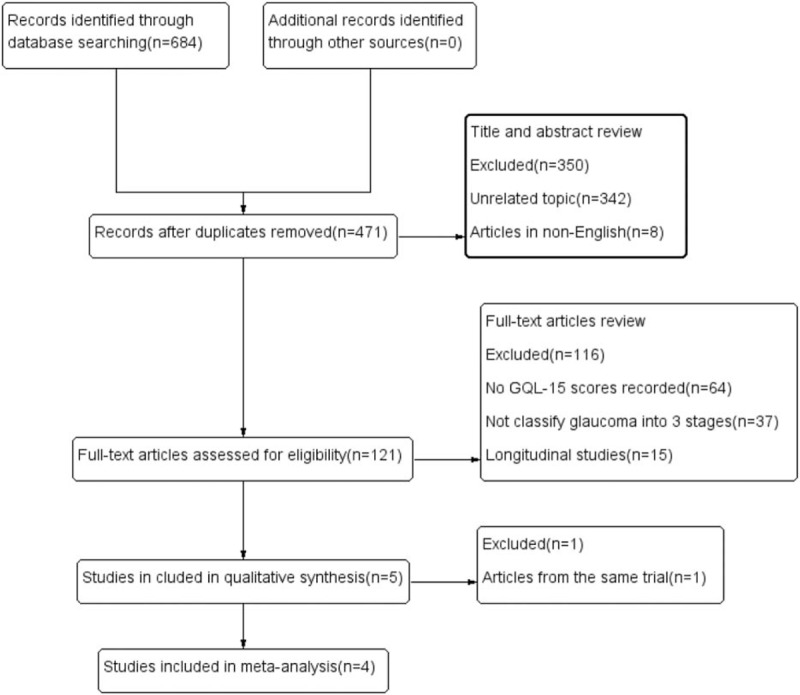
Flow diagram of the search results.

The search strategy initially identified 684 relevant articles. We reviewed the titles and abstracts of these articles, removed duplicate articles, and excluded 342 articles that were unrelated to the topic and 8 articles in languages other than English or Chinese. Based on full-text assessed, 5 articles^[9,11–14]^ met all of the inclusion criteria. Of these, 2^[12,13]^ were from the same study series; therefore, the most recent article was chosen. Ultimately, 4 studies^[9,11,13,14]^ were included in the meta-analysis. Figure 1 shows a flow diagram of the search results.

### 3.2 Characteristics and quality of the included studies

In total, 401 patients with glaucoma and 205 patients without glaucoma were included in the 4 studies analyzed, all of which were case–control studies. Three of the studies^[9,11,14]^ included a control group of healthy individuals, and the remaining^[13]^ study included a control group of patients with suspected glaucoma because of ocular hypertension or suspicious changes to the optic disc. These patients did not exhibit glaucoma, visual field loss, or sufficient optic nerve changes for a diagnosis of glaucoma, and they did not receive topical therapy. All of the studies reported disease severity, which was evaluated according to perimetric test results. The characteristics of the included studies are presented in Table 1. The quality assessment included case–control studies that used the NOS scale, as shown in Table 2. One high-quality study^[11]^ and 3 medium-quality studies^[9,13,14]^ were included in the analysis.

**Table 1:**
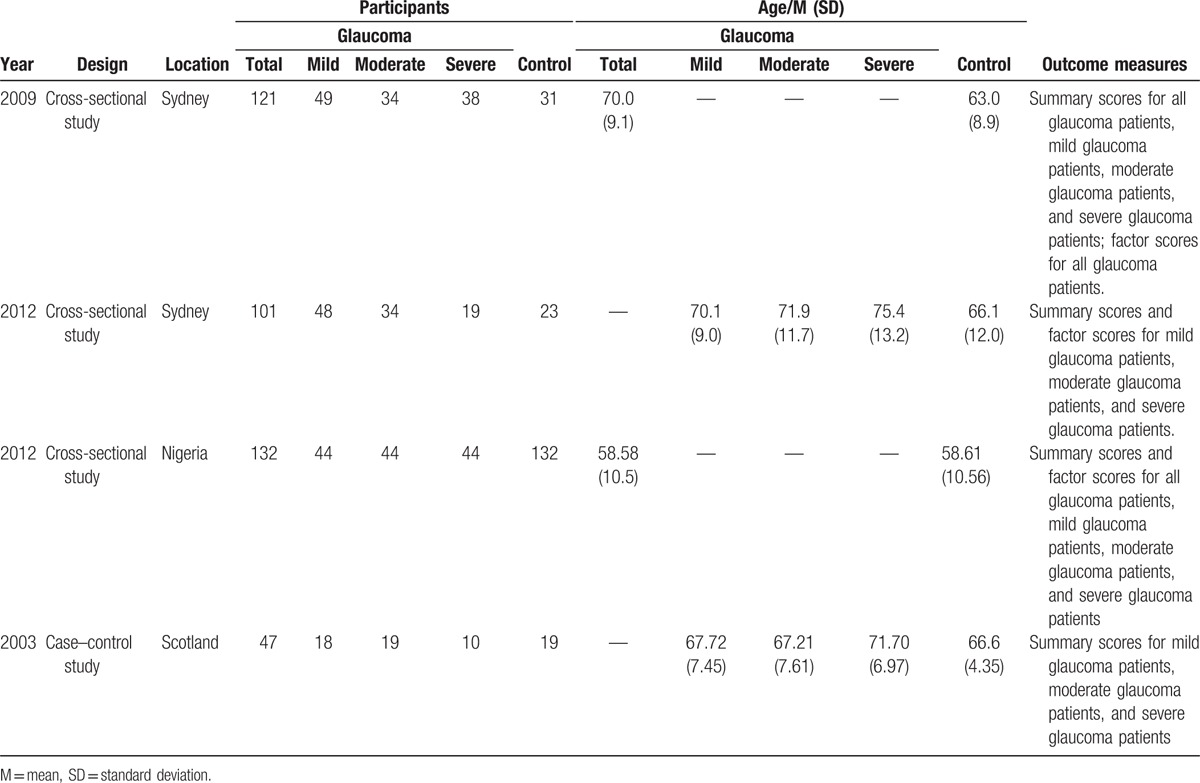
The characteristics of the included studies.

**Table 2:**
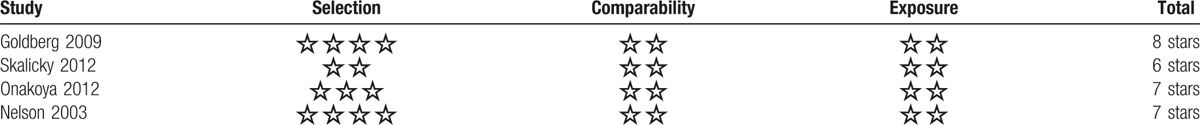
The quality assessment included case–control studies that used the NOS scale.

### 3.3 Meta-analysis of the studies with QoL-15 scores

#### 3.3.1 Comparison of QoL between patients with and without glaucoma

**Figure 2:**
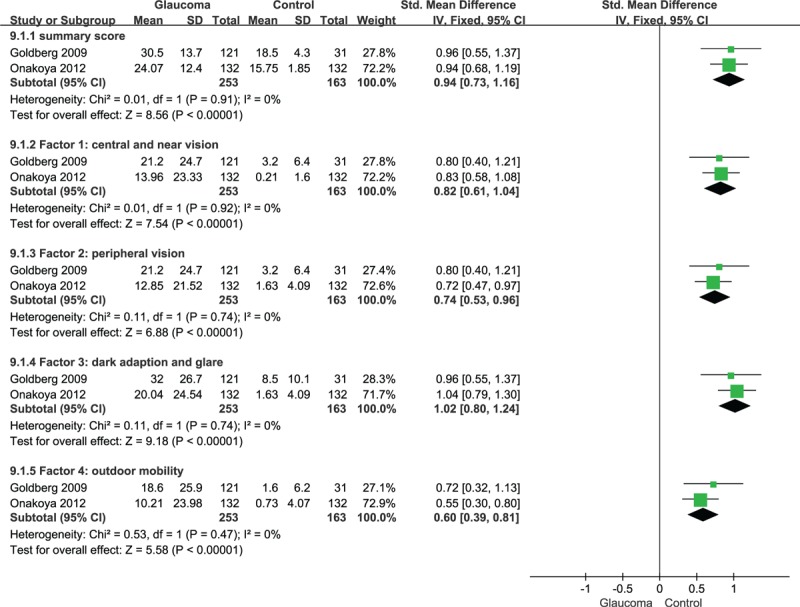
Compared the QoL of patients with glaucoma and patients without glaucoma. 95% CI = 95% confidence interval, SMD = standardized mean difference.

Figure 2 shows the results of the comparison of GQL-15 summary and factor scores between patients with glaucoma and control participants via a fixed-effects model (all *I*^2^ = 0%, *P* > .10). Patients with glaucoma showed significantly higher GQL-15 summary scores (SMD = 0.94, 95% CI = 0.73 to 1.16) and scores for all 4 factors (factor 1: central and near vision, factor 2: peripheral vision, factor 3:dark adaption and glare, and factor 4:outdoor mobility), relative to those observed for control participants (all *P* < .001). The SMDs and CIs for the 4 factor scores were as follows: central and near vision: SMD = 0.82, 95% CI = 0.61 to 1.04; peripheral vision: SMD = 0.74, 95% CI = 0.53 to 0.96; dark adaptation and glare: SMD = 1.02, 95% CI = 0.80 to 1.24; and outdoor mobility: SMD = 0.60, 95% CI = 0.39 to 0.81.

### 3.4 Comparison of QoL between patients with mild, moderate, and severe glaucoma and patients without glaucoma

**Figure 3:**
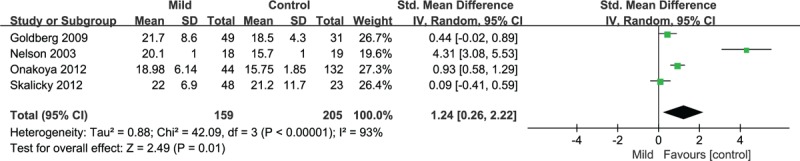
Compared the QoL of patients with mild glaucoma and patients without glaucoma. 95% CI = 95% confidence interval, SMD = standardized mean difference.

The meta-analysis was performed via the creation of a random-effects model (all *I*^2^ > 50%, *P* < .10), and the results showed that GQL-15 summary scores differed significantly between patients with mild (SMD = 1.24, 95% CI = 0.26–2.22), moderate (SMD = 2.05, 95% CI = 0.91–3.19), or severe glaucoma (SMD = 2.57, 95% CI = 1.44–3.71; all *P* < .05) and patients without glaucoma (Fig. 3, Table 3). Interestingly, the results indicated that scores for the 4 factors did not differ significantly between patients with mild glaucoma and patients without glaucoma (*P* = .11, *P* = .51, *P* = .22, and *P* = .52), but differed significantly between patients with moderate or severe glaucoma and patients without glaucoma (all *P* < .05; Table 3).

**Table 3:**
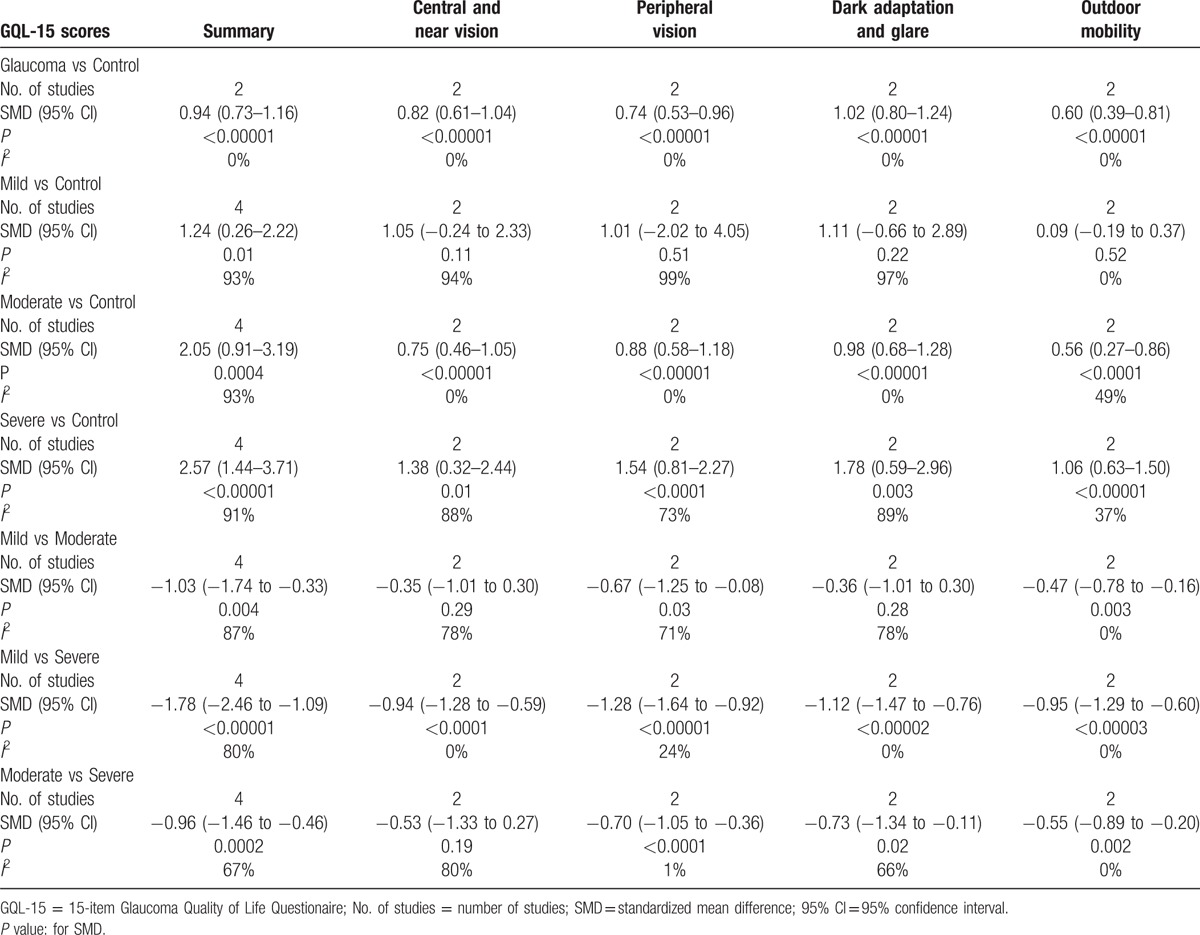
The results of this meta-analysis with QoL-15 scores.

### 3.5 Comparison of QoL between patients with mild, moderate, and severe glaucoma

#### 3.5.1 Mild versus moderate

Factor scores for central and near vision (SMD = −0.35, 95% CI = −1.01 to 0.30, *P* = .29) and dark adaptation and glare (SMD = −0.36, 95% CI = −1.01 to 0.30, *P* = .28) did not differ significantly between patients with mild and moderate glaucoma. However, summary scores (SMD = −1.03, 95% CI = −1.74 to −0.33, *P* = .004) and scores for the other 2 factors (peripheral vision and outdoor mobility, both *P* < .05) differed significantly between patients with mild and moderate glaucoma (Table 3). Only the outdoor mobility factor was included in a fixed-effects model (*I*^2^ = 0%, *P* = .88), and the remaining 3 factors and summary scores were pooled and included in a random-effects model (all *I*^2^ > 50%, *P* < .10).

#### 3.5.2 Mild versus severe

The comparison of scores between patients with mild and severe glaucoma showed that summary scores (SMD = −1.78, 95% CI = −2.46 to −1.09, *P* < .001) in a random-effects model (*I*^2^ = 80%, *P* = .002) and all of the factor scores (all *P* < .001) in a fixed-effects model (all *I*^2^ < 50%, *P* > .10) differed significantly between the 2 groups (Table 3).

#### 3.5.3 Moderate versus severe

The comparison of scores between patients with moderate and severe glaucoma showed that summary scores (SMD = −0.96, 95% CI = −1.46 to −0.46, *P* = .0002) and 3 of the factor scores (peripheral vision, dark adaptation and glare, and outdoor mobility; all *P* < .05) differed significantly between the 2 groups; however, scores for the central and near vision factor did not (SMD = −0.53, 95% CI = −1.33 to 0.27, *P* = 0.19; Table 3). The scores for the peripheral vision and outdoor mobility factors were pooled and included in a fixed-effects model (both *I*^2^ < 50%, *P* > .1). A meta-analysis of summary scores and the scores for the remaining 2 factors (central and near vision and dark adaptation and glare) was performed using a random-effects model (all *I*^2^ > 50%, *P* < .10; Table 3).

### 3.6 Sensitivity analysis

Although 3 of the studies included healthy individuals in their control groups, the remaining study^[12,13]^ included patients in whom glaucoma was suspected but had not been diagnosed; therefore, we removed the results of this study for each outcome measure, to determine the stability of the findings. This process demonstrated that there were no changes in the findings regarding the outcome measures, with the exception of the following 3 results. The results regarding the peripheral vision factor changed in the comparisons between patients with mild glaucoma and without glaucoma (SMD = −0.54, 95% CI = −1.05 to −0.02, *P* = .04) and patients with mild and moderate glaucoma (SMD = −0.38, 95% CI = −0.80 to 0.05, *P* = .08) following removal of the study's data. The results regarding the central and near vision factor in the comparison between patients with moderate and severe glaucoma also changed following removal of the study's data (SMD = −0.92, 95% CI = −1.36 to −0.48, *P* < .001).

## 4 Discussion

QoL refers to physical wellness, psychological health, and happiness. Patients with glaucoma are often most interested in factors that affect their QoL directly, such as comfort and the extent to which they are able to see, and uninterested in clinical indicators, such as IOP values and loss of the visual field, which are the issues that clinicians focus on most strongly. Glaucoma influences various aspects of patients’ lives such as their psychological well-being, economical circumstances, and comfort, which is affected by the side effects of medication, particularly with long-term treatment; in addition, visual field dysfunction affects patients’ QoL.

Several generic instruments have been developed to measure QoL. These instruments (e.g., the Short Form-36 developed for the Medical Outcomes Study)^[15]^ are sometimes used to measure vision-related QoL in ophthalmic patients. Vision-related QoL instruments are used to assess QoL in patients with various disease-specific and nonspecific eye problems.^[16]^ Nonspecific vision-related QoL instruments include the 25-item National Eye Institute Visual Activities Questionnaire,^[17]^ 14-item Visual Functioning Index,^[18]^ Visual Activities Questionnaire,^[19]^ and the Activities of Daily Vision Scale.^[20]^ Disease-specific instruments, such as the GQL-15^[9]^ and the Glaucoma Symptom Scale,^[21]^ are specifically designed for use with patients with glaucoma. The GQL-15 is a newly developed and validated questionnaire designed to assess QoL in glaucoma patients, and focuses on the visual field, which is an important factor in managing glaucoma and has been identified as an indicator of disease severity. Several articles have shown that loss of the visual field exerted a significant impact on QoL in glaucoma patients. For example, a previous study^[22]^ found that QoL underwent longitudinal changes associated with alterations in the visual field over time in patients with glaucoma. In addition, another study^[23]^ showed that the progression of loss of sensitivity in the central visual field led to reductions in QoL in patients with glaucoma. The present study sought to examine differences in QoL between patients with mild, moderate, and severe glaucoma. To this end, we performed a comprehensive literature search to identify studies that assessed QoL in patients with mild, moderate, and severe glaucoma, using the GQL-15, and conducted a meta-analysis. The results could guide clinicians in the development of treatments specific to individual patients with glaucoma of various levels of severity.

The GQL-15 questionnaire was developed to evaluate visual field loss specifically. A pilot study began with 62 items that pertained to 10 aspects of daily life; this was later reduced to 15 items that were significantly predictive of visual field loss.^[24]^ Factor analysis was used to divide the 15 items between 4 factors, as follows: 2 items associated with central and near vision (reading/recognizing faces), 6 items associated with peripheral vision, 6 items associated with dark adaptation and glare, and 1 item associated with outdoor mobility. Conjoint analysis showed the relative utility of the GQL-15 questionnaire and the 2 main priorities were identified as central vision (reading or seeing detail) and outdoor mobility (moving around outside the house).^[25]^ The GQL-15 has recently been evaluated using Rasch analysis, and the results showed excellent measurement precision and well-spaced category thresholds.^[26]^ In addition, the GQL-15 questionnaire outcomes were significantly correlated with visual field loss. Moreover, it is considered easy to understand and can be completed within a reasonable amount of time. The GQL-15 questionnaire was chosen as an assessment tool in the present study, as it has demonstrated good validity, reliability, internal consistency, and reproducibility. However, it focuses mainly on the physical effects of the disease process and does not consider broader QoL-related factors including psychological issues.

Comparison of patients with and without glaucoma in the 4 included studies showed that patients with mild, moderate, and severe glaucoma exhibited significantly poorer QoL relative to that observed in patients without glaucoma. The results also indicated that QoL declined in patients with glaucoma who experienced loss of the visual field. One study^[27]^ showed that patients’ QoL decreased in both the early and advanced stages of glaucoma. This could occur because human perception is acquired mainly via the visual system. Once vision or the range of vision is impaired, individuals feel vulnerable, which affects their work and daily lives. In the present study, QoL declined as glaucoma severity increased. However, central and near vision and dark and glare adaptation factor scores did not differ significantly between patients with mild and moderate glaucoma, whereas mean scores for the remaining 2 factors (peripheral vision and outdoor mobility) in patients with mild glaucoma were lower relative to those observed in patients with moderate glaucoma. The sensitivity analysis showed that the results regarding the comparison of peripheral vision between patients with mild with moderate glaucoma were unstable. In patients with mild glaucoma, visual field loss usually involved paracentral scotoma; however, this was larger and visual field loss was more common in the peripheral, rather than central, visual field in those with moderate glaucoma. Therefore, the results suggested that patients with moderate glaucoma experienced greater difficulty with respect to peripheral vision relative to that observed for those with mild glaucoma. Further high-quality studies should be conducted to verify the results of the present study. In addition, central and near vision did not differ significantly between patients with mild and moderate glaucoma or those with moderate and severe glaucoma. The sensitivity analysis demonstrated that the results regarding the comparison of central and near vision between patients with moderate and severe glaucoma were unstable. The reason for this finding could be that central and near vision is most important for human work and life, and once it is damaged, patients experience rapid deterioration of their QoL. However, the small size of the sample in the study could have limited the accuracy of the results.

In clinical practice, poor QoL affects patients’ confidence in long-term treatment and increases their fears and concerns regarding blindness. Ultimately, QoL continues to decline and could cause patients to cease treatment; this reflects the vicious cycle that occurs in individuals with the condition. Therefore, treatment should not focus only on clinical goals such as target IOP control, mitigation of visual field loss, and protection of the optic nerve. Moreover, clinicians should pay greater attention to patients’ QoL in the treatment and management of glaucoma. Ophthalmologists should also guide patients in obtaining knowledge regarding glaucoma, adapting to changing circumstances in their lives, and developing optimistic attitudes, and provide psychological treatment in the early stages of the disease.

The study was subject to some limitations. First, to allow the comparison of results, we included only studies in which glaucoma patients were classified into 3 groups (mild, moderate, and severe). Therefore, the meta-analysis included only 4 studies. Second, we found evidence of substantial heterogeneity when we compared GQL-15 scores. To address this issue, we created random-effects models, in which it was assumed that true effect sizes were not identical across studies but showed sufficient commonality to allow a meta-analysis. Third, we assessed study quality using only the NOS scale, and only 1 study was identified as a high-quality study. We did not assess publication bias because of the limited number of studies; however, this could have reduced the validity of our results.

In conclusion, the results showed that glaucoma led to reductions in patients’ QoL. Patients with mild glaucoma showed better QoL relative to that observed in patients with moderate or severe glaucoma. In addition, as the visual field continued to decrease, patients’ QoL declined.

## Acknowledgments

The authors thank Xiantao Zeng's (Zhongnan Hospital of Wuhan University, Wuhan, China) guidance in the use of RevMan 5.3 software.
